# TGF-β and BMP signaling in osteoblast, skeletal development, and bone formation, homeostasis and disease

**DOI:** 10.1038/boneres.2016.9

**Published:** 2016-04-26

**Authors:** Mengrui Wu, Guiqian Chen, Yi-Ping Li

**Affiliations:** 1 Department of Pathology, University of Alabama at Birmingham, Birmingham, USA; 2 Department of neurology, Bruke Medical Research Institute, Weil Cornell Medicine of Cornell University, White Plains, USA

## Abstract

Transforming growth factor-beta (TGF-β) and bone morphogenic protein (BMP) signaling has fundamental roles in both embryonic skeletal development and postnatal bone homeostasis. TGF-βs and BMPs, acting on a tetrameric receptor complex, transduce signals to both the canonical Smad-dependent signaling pathway (that is, TGF-β/BMP ligands, receptors, and Smads) and the non-canonical-Smad-independent signaling pathway (that is, p38 mitogen-activated protein kinase/p38 MAPK) to regulate mesenchymal stem cell differentiation during skeletal development, bone formation and bone homeostasis. Both the Smad and p38 MAPK signaling pathways converge at transcription factors, for example, Runx2 to promote osteoblast differentiation and chondrocyte differentiation from mesenchymal precursor cells. TGF-β and BMP signaling is controlled by multiple factors, including the ubiquitin–proteasome system, epigenetic factors, and microRNA. Dysregulated TGF-β and BMP signaling result in a number of bone disorders in humans. Knockout or mutation of TGF-β and BMP signaling-related genes in mice leads to bone abnormalities of varying severity, which enable a better understanding of TGF-β/BMP signaling in bone and the signaling networks underlying osteoblast differentiation and bone formation. There is also crosstalk between TGF-β/BMP signaling and several critical cytokines’ signaling pathways (for example, Wnt, Hedgehog, Notch, PTHrP, and FGF) to coordinate osteogenesis, skeletal development, and bone homeostasis. This review summarizes the recent advances in our understanding of TGF-β/BMP signaling in osteoblast differentiation, chondrocyte differentiation, skeletal development, cartilage formation, bone formation, bone homeostasis, and related human bone diseases caused by the disruption of TGF-β/BMP signaling.

## Introduction

The transforming growth factor-β (TGF-β) superfamily comprises TGF-βs, Activin, bone morphogenetic proteins (BMPs) and other related proteins.^1–4^ TGF-β superfamily members act through a heteromeric receptor complex, comprised of type I and type II receptors at the cell surface that transduce intracellular signals via Smad complex or mitogen-activated protein kinase (MAPK) cascade.^1–4^ At least 29 and probably up to 42 TGF-β superfamily members, five type II receptors and seven type I receptors are encoded by the human genome.^2^ Signals transduced by TGF-β superfamily members regulate the establishment of tissue differentiation through their effects on cell proliferation, differentiation, and migration.^1–4^

Fetus mammalian skeletal development begins with the condensation of mesenchymal stem cells (MSCs) from neural crest or mesoderm, and is accomplished in two distinct processes: endochondral ossification and intramembranous ossification.^5,6^ Endochondral ossification takes place in skull base and the posterior part of the skull, the axial skeleton, and the appendicular skeleton. Intramembranous ossification takes place in membranous neuro- and viscerocranium and some parts of the clavicles.^5,6^ During endochondral ossification, condensed mesenchyme undergoes chondrogenesis and forms cartilage model (anlagen), which is later replaced by mineralized bone. Differentiated chondrocytes were organized into growth plate comprised of different zones, including resting zone, proliferation zone, hypertrophic zone, and calcified zone.^5,6^ During intramembranous ossification, condensed mesenchyme directly differentiates into osteoblasts.^5,6^ Several cytokines and growth factors orchestrate skeletogenesis (for example, fibroblast growth factor (FGF), Notch, Wnt, Sonic hedgehog (SHH), Indian hedgehog (IHH), parathyroid hormone-related peptide (PTHrP), TGF-β, and BMP). Among them, TGF-βs and BMPs have diverse functions in skeletogenesis, including mesenchyme condensation, skeleton morphogenesis, growth plate development, and osteoblast differentiation.^4^ A wide variety of inheritable developmental bone diseases are caused by human genetic mutations related to TGF-β and BMP signaling.

Besides their roles in bone development, TGF-βs and BMPs also regulate the maintenance of postnatal bone and cartilage. TGF-βs have essential roles in coupling bone construction by osteoblast and bone destruction by osteoclast^7,8^ through osteoclast-mediated *Atp6i*-specific extracellular acidification^9,10^ and *Cathepsin K-*specific extracellular matrix proteins.^11,12^ Multiple BMPs are also potent osteogenic agents that possess significant clinical implication to accelerate fracture healing in patients.^13,14^ Furthermore, TGF-βs and BMPs regulate postnatal joint cartilage homeostasis, thus dysregulated TGF-β and BMP signaling are often associated with osteoarthritis in both human disease and mouse models.^15–19^

**Figure 1:**
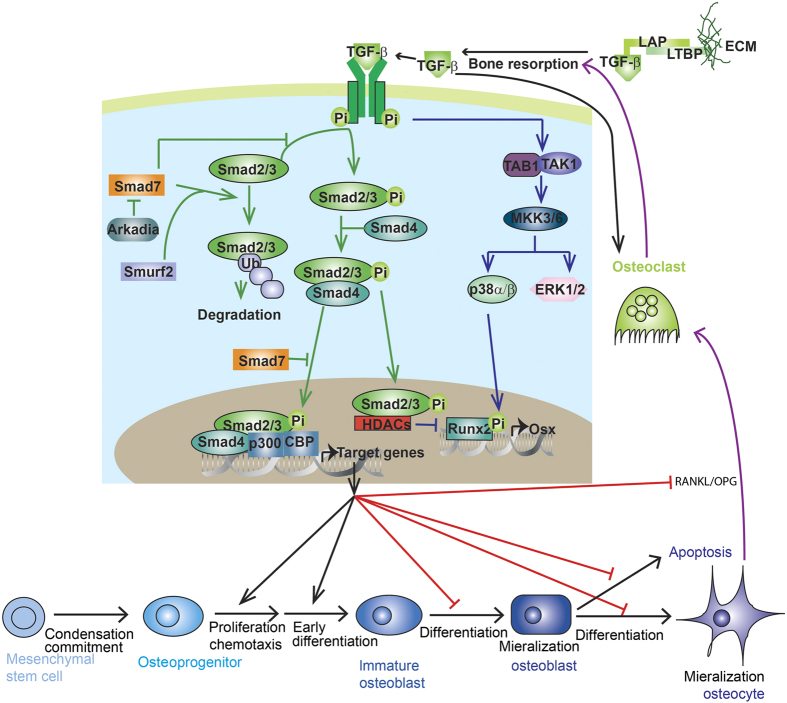
TGF-β signaling in bone. TGF-β is synthesized as a latent protein stored in the ECM, whose activation depends on osteoclastic bone resorption. Active TGF-βs binds to tetrameric receptor complex comprising two TGF-β types I receptors (TβRI) and two type II receptors (TβRII). TβRII transphosphorylases TβRI to induce Smad-dependent and non-Smad-dependent signaling. In the Smad-dependent signaling, phosphorylated R-Smad (Smad2 or 3) complexes with Smad4 and co-translocates into the nuclei, where they recruit co-factors to regulate target gene expression. In the non-Smad-dependent pathway, phosphorylated TAK1 recruit TAB1 to initiate the MKK-p38 MAPK or MKK–ERK1/2 signaling cascade. Smad7 negatively regulate Smad signaling by preventing R-Smad phosphorylation, targeting R-Smad for ubiquitin–proteasome degradation with Smurf2 and inhibiting R-smad/co-Smad complex nuclei translocation. Arkadia positively regulates Smad signaling by targeting Smad7 for ubiquitin–proteasome degradation. MAPK phosphorylates Runx2 to promote its transcriptional activity while Smad2/3 recruits HDACs to antagonize Runx2 activity. TGF-β–Smad signaling promotes proliferation, chemotaxis, and early differentiation of osteoprogenitor. However, it inhibits osteoblast maturation, mineralization, and transition into osteocyte. It also inhibits osteoclast differentiation by decreasing RANKL/OPG secretion ratio, although it promotes osteoclastogenesis via directly binding with receptors on the osteoclast. TGF-β, transforming growth factor-β.

**Figure 2:**
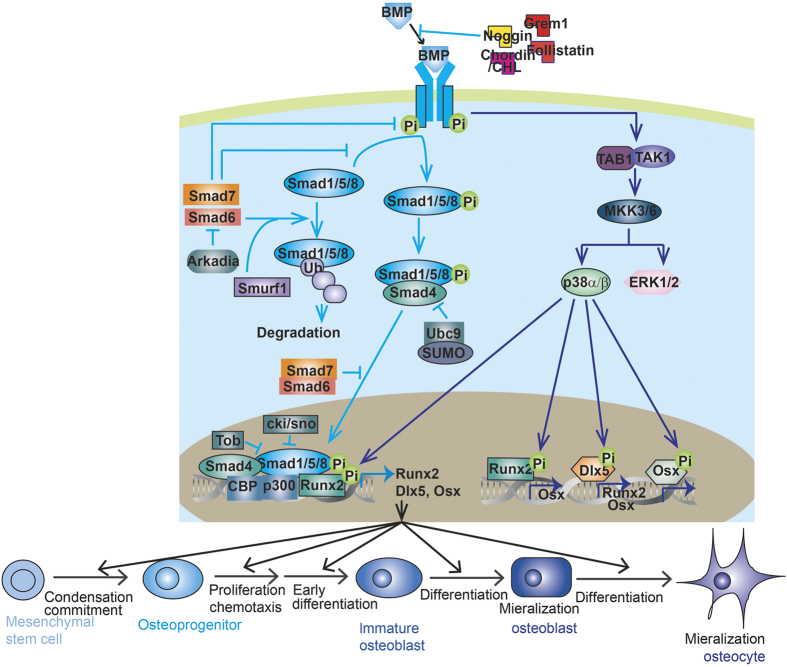
BMP signaling in bone. BMP activity is antagonized by cognate binding proteins, including Noggin, Grem1, Chordin, CHL, and Fellistatin. BMPs bind to homomeric type II receptors, which transphosphorylases homomeric type I receptor to induce Smad-dependent and non-Smad-dependent signaling. In the Smad-dependent signaling, phosphorylated R-Smad (Smad1, 5, or 8) complexes with Smad4 and co-translocates into the nuclei, where they recruit co-factors and Runx2 to regulate osteogenic gene expression, for example, Runx2, Dlx5, and Osx. In the non-Smad-dependent pathway, phosphorylated TAK1 recruit TAB1 to initiate the MKK-p38 MAPK or MKK–ERK1/2 signaling cascade. MAPK phosphorylates Runx2, Dlx5, and Osx to promote their transcriptional activity. MAPK also phosphorylates Runx2 to promote the formation of Smad–Runx2 complex. I-Smad (Smad6 or 7) negatively regulates Smad signaling by preventing R-Smad phosphorylation, targeting R-Smad or type I receptor for ubiquitin–proteasome degradation with Smurf1 and inhibiting R-smad/co-Smad complex nuclei translocation. Arkadia positively regulates Smad signaling by targeting I-Smads for ubiquitin–proteasome degradation. Ubc9/SUMO complex negatively regulates Smad signaling by targeting Smad4 for ubiquitin–proteasome degradation. BMP–Smad signaling promotes almost every step during osteoblast differentiation and maturation. BMP, bone morphogenetic protein.

In this review, we will focus on current understanding of the mechanisms by which different TGF-βs and BMPs transduce signaling and exert their functions in skeletal development and homeostasis (Figures 1 and 2). We will review mouse models (Table 1) and human bone disorders (Table 2) caused by TGF-β/BMP dysregulation. We will also discuss the crosstalk between TGF-β/BMP signaling and other signaling pathways including Wnt, Hedgehog, Notch, and FGF in bone. Those studies have opened new prospects for generating novel prognostics and therapies against bone diseases.

## The role of TGF-β signaling in bone

There are three TGF-βs: TGF-β1, TGF-β2, and TGF-β3 in mammals.^2^ TGF-βs elicit their cellular response via binding to a tetrameric receptor complex comprising two TGF-β type I receptors (TβRI/ALK5) and two type II kinase receptors (TβRII)^2^ (Figure 1). TβRII transphosphorylases TβRI resulting in subsequent phosphorylation of receptor-activated Smads (R-Smads), Smad2 and 3 (Figure 1).^2^ R-Smads then interact with the common Smad (Co-Smad), Smad4, and translocate into the nucleus, where they recruit co-factors to regulate gene (for example, CREB-binding protein (CBP) or p300) transcription.^2^ Recent studies have revealed that TGF-βs can also activate another group of R-Smad (Smad1, 5, and 8) via binding to ALK1.^17,20^ As an alternative non-Smad-dependent pathway, TGF-β also activates kinase 1 (TAK1) and TAK1-binding protein 1 (TAB1) which in turn initiates the MKKs (MAPK pathway member-encoding genes kinases)-p38 MAPK or -Erk (extracellular signal-regulated kinase) signaling cascade (Figure 1).^4^

### TGFβs ligands

All TGF-β isoforms are expressed by perichondrium and periosteum^21,22^ as well as epiphyseal growth plate^23^. However, only *Tgfb2*-null mice displayed severe skeletal abnormalities in both endochondral and intramembranous bone,^24^ while *Tgfb1-*deficient mice and *Tgfb3-*null mice had almost normal skeleton.^25–27^ Thus, TGF-β2, but not TGF-β1 or TGF-β3, is essential for embryonic skeleton development.

TGF-β has an important role in maintaining postnatal bone mass by coupling bone resorption and bone formation (Figure 1). TGF-βs are synthesized as large precursor molecules, composed of mature TGF-β and latency-associated protein (LAP).^28^ LAP remains noncovalently bound to mature TGF-β as it is secreted, rendering it inactive by masking the ECM (extracellular matrix) of many different tissues.^28^ Cleaving LAP by osteoclastic bone resorption release active TGF-β1 to induce enrichment of osteoprogenitor in the bone resorption lacunae.^7^ Tang *et al.* crossed *Tgfb1-*null mice with immunodeficient *Rag2−/−* mice to overcome the early death of *Tgfb1-*null mice.^8,25^ The *Tgfb1−/−Rag2−/−* mice showed a significant loss of trabecular bone density and reduced osteoblast number on the bone surface.^8^ A gain-of-function mutant of TGF-β1 causes Camurati–Engelmann disease (CED) in humans,^19,20^ which is characterized by diaphyseal thickening and fluctuating bone volume. This mutation results in a similar bone defect in mice (CED mice).^8^ However, TGF-β1 is unable to induce osteogenesis in mesenchymal pluripotent cells, but increases the pool of osteoprogenitors by inducing chemotaxis and proliferation.^8^ Apoptosis of osteoblasts is also blocked by TGF-β1 deletion through maintenance of survival during transdifferentiation into osteocytes.^29^ In addition, TGF-β treatment blocked osteoblast mineralization in culture,^30^ indicating its bi-functions in osteoblast differentiation. On the other hand, active TGF-βs regulate bone resorption in a dose-dependent manner. The gradient of TGF-β created during osteoclast bone resorption can limit further osteoclast activity.^28^ Low concentrations of active TGF-β can induce macrophage migration to the bone resorption pits, whereas high concentrations of active TGF-β inhibit migration of osteoclast precursors.^28^ TGF-β was also shown to promote osteoclast differentiation at low dose and inhibits osteoclast differentiation at high dose through regulating RANKL/OPG ratio secreted by osteoblasts.^7,31^ Furthermore, TGF-β1 via binding to its receptor on osteoclasts activates Smad2/3, which associates directly with TRAF6–TAB1–TAK1 complex and favors osteoclast differentiation.^32^

TGF-β is a double-edged sword in the maintenance of articular cartilage metabolic homeostasis and the pathogenesis of arthritis^33^. Loss of TGF-β signaling in cartilage induces chondrocyte hypertrophy, ultimately resulting in cartilage degeneration, and pharmacological activation of the TGF-β pathway has therefore been proposed to preserve articular cartilage integrity during osteoarthritis (OA).^19,34,35^ However, age-dependent switch from TGF-β-ALK5-Smad2/3 to -ALK1-Smad1/5/8 signaling contributes to osteophyte formation and the pathogenesis of osteoarthritis.^17,36^ At the onset of either OA or rheumatoid arthritis (RA), elevated active TGF-β1 resulted from excessive bone resorption recruits MSCs in the subchondral bone marrow and induces the formation of “osteoid islet”, which leads to the degeneration of the overlying articular cartilage.^37,38^ Transgenic expression of active TGF-β1 in osteoblastic cells induced OA, whereas inhibition of TGF-β activity in subchondral bone attenuated the degeneration of articular cartilage.^37^ Similarly, aberrant activation of TGF-β in subchondral bone is involved at the onset of RA joint cartilage degeneration, whereas either systemic or local blockade of TGF-β activity in the subchondral bone attenuated articular cartilage degeneration in RA.^38^ Furthermore, knockout of the TβRII in nestin-positive MSCs leads to less development of both OA and RA relative to wild-type mice.^37,38^

### Extracellular regulation of TGF-β signaling

ECM proteins regulate TGF-β signaling through controlling ligand availability (Figure 1). LTBPs covalently bind latent TGF-β and modulate tissue levels of TGF-β. Ablation of LTBP-3 reduces both osteogenesis and bone resorption, and resulted in increased bone mass associated with decreased levels of TGF-β.^39,40^ Binding of TGF-β to small leucine-rich proteoglycans (decorin, biglycan, and lumican) restricts the TGF-β in the ECM so as to inhibit its activity.^41^ *Bgn* and *Dcn* double deficiency results in osteopenia and a striking change in collagen fibril shape.^41^ Excessive TGF-β signaling, caused by reduced binding of decorin, is also found to be a common mechanism of osteogenesis imperfecta in mouse models.^42^

### TGF-βs receptors

Similarly to TGF-βs, the expression of TGF-β receptors is also detected in the growth plate and perichondrium.^21,22,43^ Mice lacking either TβRI/ALK5 or TβRII in MSC results in short long bone and defects in joint development fusion.^30,44–46^ MSC-specific *Tgfbr2-*deletion and MSC-specific *Alk5-*deletion both promote chondrocyte hypertrophy and decreased chondrocyte proliferation, which was also observed in DN-TβRII transgenic mice.^30,34,44–46^ Thus, TGF-β signaling favors chondrocyte proliferation but blocks the transition from proliferative chondrocyte into hypertrophic chondrocyte. Mice lacking TβRII/ALK5 in the chondrocyte can reproduce defects in the skull base and vertebrae as those observed in the *Tgfb2-*null mice,^24,47^ but cannot influence normal long bone development.^47^ This phenotype indicates that TGF-β might regulate growth plate development largely through mediating paracrine cytokines secretion by perichondrium cells.

TGF-β signaling favors bone formation by promoting osteoprogenitor enrichment. Consistently, deletion of *Alk5* in MSC resulted in reduced bone collars and trabecular bones associated with decreased osteoblast number.^30^ The study showed that TGF-β, through TβRI, promotes pre-osteoblast commitment and early differentiation.^30^ However, deletion of *Tgfbr2* in osteoblasts resulted in an unexpected increase in bone mass due to enhanced PTHrP signaling.^43^

### Smad-dependent pathway

Smad2 and Smad3 respond to TGF-β and regulate TGF-β-mediated osteoblast and chondrocyte differentiation (Figure 1). Smad2/3 inhibits Runx2 expression, and activated Smad3 also recruits class II histone deacetylases (HDACs) 4 and 5 to repress the function of Runx2.^48–51^ TGF-β can no longer inhibit the differentiation of osteoblasts in the absence of Smad3.^52,53^ Although TGF-β-Smad3 negatively regulates osteoblastogenesis, it also inhibits osteoblast apoptosis and the differentiation into osteocyte. Thus, *Smad3-*null mice are osteopenic associated with increased osteocyte number and apoptosis.^52^ Furthermore, TGF-β-induced inhibition of chondrocyte maturation is potentiated by Smad2/3 signaling,^54^ and abolished by disrupting Smad2/3 activation.^54,55^ *Smad3*-null mice also displayed accelerated chondrocyte hypertrophy, at least partially through reduced Noggin level and unopposed BMP signaling.^56^

In addition, TGF-β-Smad3 signaling is required for postnatal maintenance of articular cartilage homeostasis. *Smad3*-null mice and chondrocyte-specific *Smad3* CKO mice exhibit articular cartilage degeneration and automatically develop OA.^57–59^ Mutations of Smad3 in human caused aneurysms–osteoarthritis syndrome,^15,60,61^ which is presenting with aneurysms, mild craniofacial features, and skeletal and cutaneous anomalies. Mechanistically, TGF-β signals through Smad3 to confer a rapid and dynamic repression of Runx2-induced MMP-13 expression, and signals through p38 to promote Runx2-induced MMP-13 expression in the absence of Smad3.^58^

### Non-Smad-dependent pathway

Non-Smad-dependent pathway initiated by TGF-β also contributes to bone formation. Studying MSC-specific *Alk5* CKO mouse model revealed that TGF-β promotes osteoblast proliferation and early differentiation through both Smad2/3 and MAPK signaling pathway.^30^ MAPK signal activated by TGF-β and BMP positively regulates Runx2 expression and function to promote MSC differentiation.^62^ Furthermore, TGF-β2 stimulates cranial suture closure by promoting osteoprogenitor proliferation via the MKK–ERK signaling.^63^

Previous studies showed that TGF-β, through Alk5-Smad2/3 or Alk5-MAPK signaling, promotes osteoblast progenitor enrichment and early differentiation, but negatively regulates osteoblast differentiation and mineralization at latter stages. TGF-β is also a molecule coupling bone formation with bone resorption. The relationship between TGF-β and cartilage is more complex. Either loss of TGF-β or excessive active TGF-β contributes to the progress of osteoarthritis. Further studies will elucidate how TGF-β dose-dependently and stage-dependently regulates cartilage integrity. Those findings would facilitate the design of therapeutic approaches for targeting TGF-β in OA treatment.

## The role of BMP signaling in bone

BMP signaling is mediated through type I and type II BMP receptors (Figure 2). After binding to BMP ligands, homomeric dimers of the type II receptors form a tetrameric complex with homomeric dimers of the type I receptors, and induce transphosphorylation of the type I receptors. This dynamic interaction leads to signal transduced through either Smads or MAPKs, which further activates the transcription of specific target genes involved in osteoblastic differentiation and bone formation (Figure 2).

### BMP ligands

Among the 14 BMPs, BMP-2, 4, 5, 6, 7, and 9 exhibit high osteogenic activity.^64,65^ Osteogenic capability of BMP-2 and BMP-7 have been vastly studied and the recombinant proteins are currently being investigated in human clinical trials of craniofacial deformities, fracture healing, and spine fusion.^13,14^ BMP-2 vastly increases osteocalcin expression^66^ and a short-term expression of the BMP-2 is necessary and sufficient to irreversibly induce bone formation.^67^ BMP-7 induces the expression of osteoblastic differentiation markers, such as ALP and accelerates calcium mineralization.^68–70^ BMP-3 is a ‘non-canonical’ BMP molecule that transduces type IIB Activin receptor (AcvrIIB)-Smad2/3 signaling to oppose the osteogenic function of other BMPs.^71^ *In vivo*, BMP-3 is mainly produced by osteoblasts and osteocytes,^71^ and negatively regulates bone mass *in vivo*, as shown by knockout and transgenic mouse models.^72,73^ Interestingly, a recent study showed that BMP2 addition to culture media rapidly induced expansion of isolated mouse skeletal stem cell (mSSC).^74^ Delivery of BMP2 induces *de novo* formation of the mSSC and bone in extraskeletal locations.^74^ In addition, co-delivery of BMP2 and sVEGFR1 induces *de novo* formation of cartilage in extraskeletal locations.^74^ Expression of the BMP antagonist gremlin 1 defines a population of osteochondroreticular stem cells in the bone marrow, which is self-renewal and is able to generate osteoblasts, chondrocytes, and reticular marrow stromal cells, but not adipocytes.^75^

BMPs have essential roles at many steps in endochondral bone development. Studies showed that BMP signaling promotes chondrocyte proliferation and differentiation,^76–78^ by maintaining the expression of Sox9, a transcription factor that is essential for chondrogenic commitment and differentiation.^77,79^ BMP-7 null mice died shortly after birth and exhibits skeletal patterning defects restricted to the rib cage, skull, and hindlimbs.^80^ Conditional deletion of BMP-2, BMP-4, or BMP-7 in MSC results in normal skeletongenesis.^70,81,82^ Only BMP-2 CKO mice have frequent fractures that fail to heal, which is not observed in either *Bmp-4* or *Bmp-7* CKO mice.^70,83,84^ The studies indicate that BMPs have duplicate functions in skeleton development, while BMP-2 has a unique role in postnatal bone formation. MSC-specific *Bmp-2/-4* DKO mice have extremely malformed limbs and a severe impairment of osteogenesis, which is not observed in *Bmp-2/-7* DKO mice.^82,85^ Chondrogenic condensations in some skeletal elements fail in MSC-specific *Bmp-2/-4* DKO mice.^82^ Furthermore, chondrocyte-specific *Bmp-2* CKO and *Bmp-2/4* DKO mice exhibits severe disorganization of chondrocytes within the growth plate region.^86^ Collectively, these studies revealed that coordinated functions of endogenous BMP-2 and BMP-4 are required for osteoblastogenesis as well as chondrocyte proliferation, differentiation and apoptosis during skeletal development.

BMP7 has a pivotal role in postnatal maintenance of articular cartilage. Intra-articular administration of rhBMP7 significantly inhibited articular cartilage degeneration and blocked production of inflammatory cytokines by the synovial membrane.^18,87,88^ *Bmp7*^*f/f*^*Prx1-*Cre mice, and *Bmpr1a*^*f/f*^*Gdf5*-Cre mice exhibit articular cartilage degeneration and automatically develop OA.^89^

### BMP receptors

There are three type I receptors for BMPs, type IA BMP receptor (BMPR1A/ALK3), type IB BMP receptor (BMPR1B/ALK6), and type I activin receptor (AcvR1/ ALK2). BMPs exert their osteogenic signaling through those receptors. Activating mutants of *Acvr1* cause ectopic ossification in mouse^90^ and in human disease (fibrodysplasia ossificans progressive, FOP).^91,92^ Mechanically, active AcvR1 induces epithelial-to-mesenchymal transition and promotes ectopic osteoblastogenesis via canonical Smad1/5 signaling.^90,93^ On the other hand, blocking BMP signaling through overexpression of DN-BMPRII or deletion of *Bmpr1a* in osteoblast results in low bone mass.^94,95^ Although deletion of *Bmpr1a* in preosteoblasts causes an unexpected increase in bone mass due to reduced bone resorption, osteoblast differentiation remains low in this condition.^96–98^

**Figure 3:**
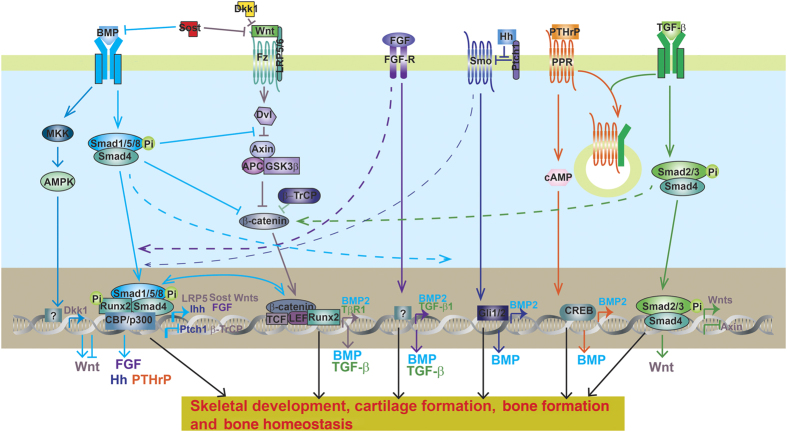
Crosstalk between BMP, TGF-β, and other signaling during osteoblast differentiation. BMP has dual roles in Wnt signaling. On one hand, BMP inhibits Wnt/β-catenin signaling by increasing Wnt antagonist Dkk1 and Sost expression and by preventing β-catenin nuclei translocation. On the other hand, BMP promotes Wnt/β-catenin signaling by forming co-transcriptional complex with β-catenin/TCF/LEF/Runx2, by increasing Wnt expression, by antagonizing Dvl function and by decreasing b-TrCP expression. BMP signaling promotes FGF, Hh, PTHrP signaling by increasing IHH expression, increasing FGF expression, decreasing Ptch1 expression, respectively. BMP is essential for IHH-induced osteoblast differentiation. FGF, IHH, Wnt, and PTHrP signaling all promotes BMP2 expression so as to enhance BMP signaling. FGF and IHH signaling is essential for BMP-induced osteoblast differentiation. TGF-β antagonizes PTHrP signaling through TGF-β type II receptor complexing and internalizing together with PTHrP receptor (PPR). TGF-β promotes Wnt activity by increasing Wnt ligands expression and decreasing Axin expression. Fibroblast growth factor (FGF) and Wnt all increase TGF-β expression to promote TGF-β signaling. BMP, bone morphogenetic protein; PTHrP, parathyroid hormone-related peptide; TGF-β, transforming growth factor-β.

The three type I BMP receptors (BMPR1A, BMPR1B, and ALK6) have largely redundant roles in skeletal development. BMPR1A and BMPR1B share similar expression pattern in chondrocyte condensations and developing skeletons,^99–104^ and AcvR1 is expressed throughout the growth plate.^105^ Activation and dominant-negative experiments proved the BMPRIB is essential for cartilage condensation in chicks.^103^ Activation experiments showed that BMPR1A, BMPR1B, and AcvR1 are all able to promote chondrogenesis.^77,101,103,106–108^ However, deletion of each of them alone only results in mild skeletal defects or defects restricted to certain skeletal elements.^77,104,105,109,110^ In contrast, *Bmpr1a*/*Bmpr1b* DKO mice, *Acvr1*/*Bmpr1a* DKO mice, and *Acvr1*/*Bmpr1b* DKO mice exhibit generalized chondrodysplasia that is much more severe than any of the corresponding mutant strains.^104,105,110^ The loss of both BMPR1A and BMPR1B blocks chondrocyte condensation, proliferation, differentiation, survival, and function due to impaired Sox proteins (Sox-5, 6, and 9) expression.^104,110^ In conclusion, those mouse models demonstrated that BMP signaling is essential for almost every step during endochondral bone development (Figure 3).

### Smad-depedent pathway

Most BMPs activate Smad1/5/8 as their R-Smad (Figure 2). Smad1/5/8-Smad4 complex transcribed Runx2 expression, as they complex with Runx2 to initiate other osteoblast gene expression.^62,111,112^ Smad1 is an important mediator of BMP’s osteogenic function. In osteoblast-specific Smad1-CKO mice, BMP signaling was partially inhibited, osteoblast proliferation and differentiation were impaired and mice developed an osteopenic phenotype.^113^ Smad1 and Smad5 together mediate the role of BMP in endochondral bone development. Chondrocyte-specific deletion of Smad1 results in delayed calvarial bone development and shortening of the growth plate.^113,114^ The addition of Smad5 haploinsufficiency or insufficiency leads to more severe chondrodysplasia, while addition of Smad8 insufficiency did not worsen the defects.^114,115^ Thus, Smad8 contributes much less to skeleton development as compared with Smad1 and Smad5. Only BMP3 also activates Smad2/3, which antagonized osteogenic activity of Smad1/5/8 and opposed the function of other BMPs in osteoblast differentiation.^116^

Both BMPs and TGF-βs signal through Smad4. Smad4 mutation in humans causes Myhre syndrome, a developmental disorder, characterized by short stature and facial dysmorphism.^117,118^ *In vitro* Smad4 ablation partially suppressed BMP-4-induced osteoblast differentiation.^119^ *In vivo* abrogation of Smad4 in chondrocytes results in dwarfism with a severely disorganized growth plate and ectopic bone collars in perichondrium.^120^ In the Smad4-deficient growth plate, resting zone was expanded, while chondrocyte proliferation was reduced and hypertrophic differentiation was accelerated.^120^ Deletion of Smad4 in mature osteoblasts causes lower bone mass and decreased osteoblast proliferation and differentiation up to 6 months of age, while the trabecular bone volume in the mutant mice increased after 7-month-old due to reduced bone resorption.^121^ Embryonic deletion of Smad4 in pre-osteoblast causes stunted growth, spontaneous fractures and a combination of features seen in osteogenesis imperfecta, cleidocranial dysplasia, and Wnt-deficiency syndromes.^122^ Postnatal deletion of Smad4 in pre-osteoblasts increases mitosis of cells on trabecular bone surfaces as well as in primary osteoblast cultures, while delays differentiation and matrix mineralization by primary osteoblasts associated with altered β-catenin acvitity.^123^ Knockout of Smad4 in osteoblast also reveals that BMP/TGFβs coordinate with Wnt signaling to maintain normal bone mass. In summary, Smad4 has multiples roles in growth plate development and postnatal bone homeostasis.

### Non-Smad-dependent pathway

TAK1–MKK–MAPK pathway is also involved in the fine-tuning of BMP effects on skeletal development and osteogenic differentiation (Figure 2). Morphological defects of BMP-receptor-deficient mice are found to be associated with a decrease in both p38 MAPK and Smad activity.^105^ Ectopic ossification of FOP patients is associated with an increase in both p38 MAPK and Smad activity.^124^ Either chondrocyte- or MSC-specific *Tak1*-deletion results in disorganized growth plates as well as a failure to maintain interzone cells of the elbow joint.^125^ Postnatal chondrocyte-specific Tak1 deletion results in severe defects of growth plate and articular cartilage development with reduced expression of SOX protein, proteoglycan and type II collagen.^126^ Osteoblast-specific deletion of Tak1 results in clavicular hypoplasia and delays fontanelle fusion, which is a phenotype similar to that of cleidocranial dysplasia, a human disease that is caused by Runx2 deficiency.^127^ Mice with deletion of other MAPK pathway member-encoding genes, MAPK kinase 3 (Mkk3), Mkk6, p38a, or p38b, also display profoundly reduced bone mass secondary to defective osteoblast differentiation.^127^ Mechanistically, MAPKs phosphorylate a number of osteoblast master transcription factors including Runx2,^111,127,128^ Dlx-5^129^ and Osterix^130–132^ (Figure 2). The phosphorylation facilitates the recruitment of cofactors and promotes their transcriptional activity.^111,127–130^ MAPKs also positively regulate Runx2 and Osterix expression to promote MSC differentiation.^62^ Furthermore, MAPK signaling enhances the canonical BMP–Smad signaling by promoting the interaction between Runx2 and Smad complex.^111^

In conclusion, most BMP ligands are strong osteogenic agents, through both Smad and non-smad signaling pathway, which synergize at osteogenic transcriptional factors (for example, Runx2, Osx). BMPs also have critical roles in multiple stages of endochondral bone development, including mesenchyme condensation, chondrocyte proliferation, and chondrocyte differentiation. In addition, BMP7 and BMPR1A also have roles in postnatal homeostasis of articular cartilage. Recent studies have shown that BMP2 enhances proliferation and enrichment of mouse skeletal stem cell. Thus, it is of full potential to utilize BMP locally in bone and cartilage regeneration.

## Regulation of BMP and TGF-β signaling in bone

BMP and TGF-β signaling are delicately controlled by multiple machineries: Extracellular matrix proteins control the availability of the ligands; Inhibitory Smads antagonize various steps in the Smad-dependent signaling; ubiquitin–proteasome machinery controls stability of various signal transducers and inhibitors; miRNAs regulate the signaling at post-translational level; co-repressors and epigenetic factors regulate the signaling at transcriptional level.

### Regulators in the ECM

ECM proteins regulate TGF-β signaling through controlling ligand availability (Figure 1). LTBPs covalently bind latent TGF-β and modulate tissue levels of TGF-β. Ablation of LTBP-3 reduces both osteogenesis and bone resorption, and resulted in increased bone mass associated with decreased levels of TGF-β.^39,40^ Binding of TGF-β to small leucine-rich proteoglycans (decorin, biglycan, and lumican) restricts the TGF-β in the ECM so as to inhibit its activity.^41^ *Bgn* and *Dcn* double deficiency results in osteopenia and a striking change in collagen fibril shape.^41^ ExcessiveTGF-β signaling caused by reduced binding of decorin is also found to be a common mechanism of osteogenesis imperfecta in mouse models.^42^

BMP signaling is regulated by a group of cognate binding proteins (for example, Noggin, Chordin, Gremlin, and Follistatin) that competitively bound BMPs to prevent their binding to receptors^133–135^ (Figure 2). Noggin opposes BMP’s osteogenic function *in vivo*^136^ and *in vitro.*^137^ *Nog*-null mice died at birth with severe malformed skeletons due to unopposed BMP signaling,^138,139^ and addition of deficiency of other BMP antagonists (Grem1 or Follistatin) aggravated the skeleton malformation of *Nog*−/− mice.^140,141^ Ectopic expression of Noggin in osteoblast impairs osteoblastogenesis and results in osteopenia in mice.^142,143^ Ectopic expression of Noggin is also able to block cartilage development, change skeletal morphology, and prevent cranial suture fusion.^144,145^ Noggin expression is induced by BMPs, and acts in a negative regulatory loop to inhibit BMP activity.^133^ Other signals including Sox9 and FGFs also regulate BMP signaling via manipulating Noggin expression.^145–148^ Chordin and Chordin-like proteins (CHL) were identified as a factor dorsalizing the Xenopus embryo.^149–152^ Chordin and CHL treatment prevented BMP-induced osteoblast differentiation and mineralization,^152,153^ as well as BMP-induced chondrocyte maturation.^149,152,154^ Furthermore, *Chordin−/−Nog+/−* mice exhibit defects restricted to the head associated with increased BMP activity.^155^

### I-Smad

Inhibitory Smads (I-Smad, Smad6, and 7) inhibits BMPs and TGF-βs signal in multiple ways: preventing R-Smad nuclei translocation, competitively binding with type I receptor to prevent R-Smad phosphorylation, and promoting R-smads or receptor degradation by recruiting E3 ubiquitin ligases Smurf1/2.^156,157^ Smad6 preferentially inhibits BMP signaling (Figure 2), while Smad7 interferes with both BMP and TGFβ signaling^157^ (Figures 1 and 2). Smad6 and 7 overexpression blocks BMPs-induced osteoblast and chondrocyte differentiation *in vitro*,^107,158–161^ and vice versa.^160^ Chondrocyte-specific Smad6 transgenic mice show postnatal dwarfism, osteopenia, and delayed chondrocyte hypertrophy due to inhibition of Smad1/5/8 signaling.^162^ Besides its role in BMP signaling, Smad6 and Smurf1 also induce Runx2 degradation in an ubiquitin-proteasome-dependent manner, which directly inhibits osteoblast differentiation.^163^ Thus, Smad6 expression is also mediated by BMP–Smad1–Runx2 signaling at transcriptional level and regulates BMP and Runx2 activity in a negative feedback loop.^160,164^ Conditional-overexpression of Smad7 in MSC or stage-specific chondrocytes results in defective mesenchymal condensation, chondrocyte proliferation and chondrocyte maturation, respectively.^165^

### Ubiquitin–proteasomal degradation pathway

Stability of protein transducers within TGFβ and BMP signaling is under control of ubiquitin enzymes and de-ubiquitin enzymes (Figures 1 and 2). Smurf2 is an E3 ubiquitin ligase that targets TGFβ receptor and Smad2/3 for ubiquitin–proteasomal degradation.^166,167^ Ectopic overexpression of Smurf2 in condensing chondrogenic mesenchyme of chicken wing bud accelerates chondrocyte maturation and ossification.^166^ Smurf1 is also an E3 ubiquitin ligase with a wide range of a targets, including BMP type I receptors, Smad1/5, Runx2, and MKK2.^162,163,167,168^ Smad6/Smurf1 double transgenic pups exhibit delayed endochondral bone formation that is more severe than Smad6 transgenic pups.^162^ Smurf1-deficient mice were born normal but exhibited a temporal increase of bone mass as they aged, due to accumulation of phosphorylated MKK2 and activation of the downstream JNK signaling cascade.^167^ Arkadia, an E3 ubiquitin ligase that induces ubiquitylation and proteasome-dependent degradation of TGF-β and BMP suppressors, such as Smad6, Smad7, and c-Ski/SnoN, promotes osteoblast mineralization and differentiation induced by BMPs.^169^ SUMO (small ubiquitin-related modifier) and Ubc9 (ubiquitin conjugating enzyme 9) target Smad4 for degradation and inhibit osteoblastic differentiation induced by BMP2.^170,171^ Furthermore, deubiquitylating enzyme USP15 mediates K48-linked deubiquitylation of ALK3, to promote ALK3-smad1 signal and BMP-induced osteoblast differentiation.^172^

### Transcriptional repressors

A group of co-repressors controls transcriptional activity of the R-Smad/co-Smad complex (Figure 2). Ski/SnoN interacts with R-Smad proteins to repress their activity. c-Ski/SnoN, evoked by TGF-βsuppresses the BMP signaling and hypertrophic chondrocyte maturation. SnoN is highly expressed in articular cartilage and may involve in TGFβ-mediated cartilage homeostasis. Tob as a co-repressor is another negative regulator of BMP/Smad signaling in osteoblasts.^173^ BMP2-signal is elevated in the absence of Tob and repressed by overproduction of Tob.^174,175^ Mice carrying a targeted deletion of the Tob gene had a greater bone mass resulting from increased numbers of osteoblasts and were protected from OVX-induced osteoporosis.^174,175^

### MicroRNAs

Recent researches have raised the importance of microRNA (miRNA) in skeleton homeostasis and in TGF-β and BMP signaling pathway (Figures 1 and 2). BMP treatment regulates multiple miRNA expression during osteoblastogenesis, and a number of those miRNAs feedback to regulate BMP signaling:^176–179^ miR-133 targets Runx2 and Smad5 to inhibit BMP-induced osteogenesis;^176^ miR-30 family members negatively regulate BMP-2-induced osteoblast differentiation by targeting Smad1 and Runx2;^177,178^ miR-322 targets Tob and enhances BMP response.^179^ In addition, some non-BMP-regulated miRNAs also have regulatory roles in BMP signaling: miR-141 and -200a remarkably modulate the BMP-2-induced pre-osteoblast differentiation through the translational repression of Dlx5;^180^ miR-542-3p targets BMP7 and represses BMP7-induced osteoblast differentiation and survival;^181^ miR-20a promotes osteogenic differentiation through upregulation of BMP/Runx2 signaling by targeting PPARγ, Bambi, and Crim1;^182^ miR-140 targets a mild inhibitor of BMP Dnpep, and loss of miR-140 in mice causes growth defects of endochondral bones and craniofacial deformities.^183^ Furthermore, several miRNAs are reported to regulate chondrocyte differentiation by targeting TGF-β and BMP signaling. Profiling identified expression of several miRNAs that changed significantly in human OA chondrocyte compared with normal cells targets Smad-signaling, including miR-20b, miR-146a, and miR-345.^184–186^ miR-146a, a miRNA increased in OA, targets Smad4 and induces chondrocyte apoptosis and cellular responsiveness to TGF-β.^187,188^ miR-199a(*) targets Smad1 and adversely regulates early chondrocyte differentiation.^189^

### Epigenetic regulation

The Sox9-related transcriptional apparatus activates its target gene expression through p300-mediated histone acetylation on chromatin, and TGF-β has an important role in recruiting p300 to the promoter.^190^ TGF-β also induces the expression of KDM4B, which removes the silencing H3K9me3 marks on the Sox9 promoter and increased Smad3 occupancy on the Sox9 promoter, so as to enhance Sox9 expression.^191^ HDAC inhibitor suberoylanilide hydroxamic acid (SAHA; vorinostat) increases the Runx2 promoter acetylation to intrigue Runx2 expression and osteoblastogenesis, in a *BMP2-*dependent manner.^192^ The *Bmp2* promoter regions of the genes are epigenetically locked with increased CpG methylation and decreased H3K9 acetylation.^193^ And CpG-demethylating agent or the HDAC inhibitor trichostatin-A renders Bmp2 expression.^193^ At the protein level, HDACs deacetylate Smad7 to protect it against ubiquitination and degradation.^4^ BMP-2 signaling stimulates p300-mediated Runx2 acetylation, which increases its stability and transactivation activity.^4^ HDAC4 and HDAC5 deacetylate Runx2 and renders its degradation through ubiquitin–proteasomal pathway.^4^

In summary, traditional regulators (for example, ECM, I-Smad, ubiquitin-related proteins, transcriptional co-factors) of BMP and TGF-β signaling have been extensively studied and the regulatory network is finely established. Prosperous progress will be made to discover novel regulation of BMP and TGF-β signaling by miRNA and epigenetic factors, and will provide more clues to design new treatment for bone diseases through targeting BMP and TGF-β signaling.

## Crosstalk between TGF-β and BMP signaling with other signaling in bone

**Figure 4:**
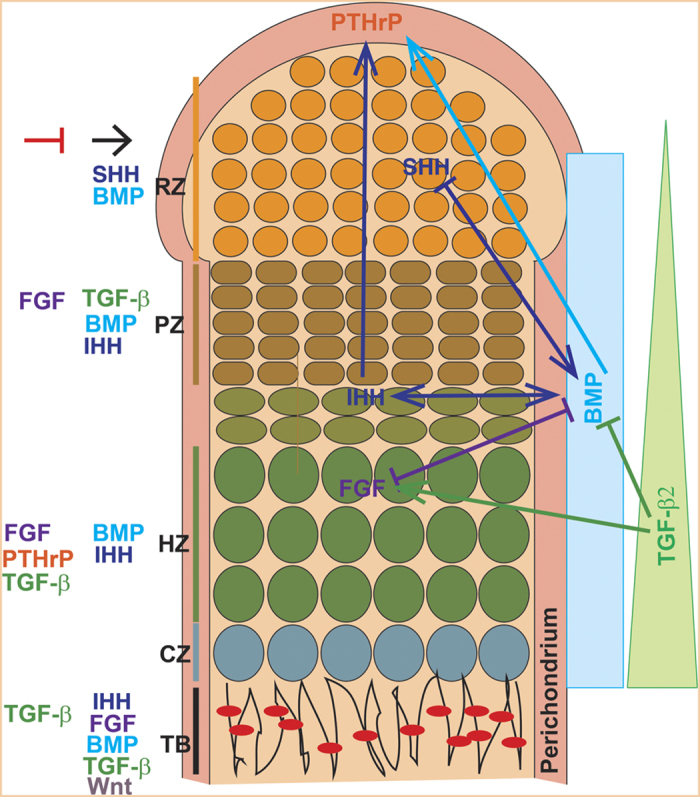
Crosstalk between BMP, TGF-β and other signaling in the growth plate. In the epiphyseal growth plate, differentiating chondrocytes are organized into four layers, including resting zone (RZ), proliferation zone (PZ), hypertrophic zone (HZ), and calcified zone (CZ). The calcified zone was gradually replaced by the trabecular bone (TB). Positive regulating cytokines (→) and negative regulating cytokines (--**|**) of each zone are listed on the left adjacent to them. BMP, bone morphogenetic protein; FGF, fibroblast growth factor; IHH, Indian hedgehog; PTHrP, parathyroid hormone-related peptide; SHH, Sonic hedgehog; TGF-β, transforming growth factor-β.

Coordinated signaling networks controlled by multiple cytokines regulate skeletogenesis and osteoblast differentiation. To regulate bone homeostasis, TGF-β and BMP signaling dynamically crosstalks with other pathways including Wnt, Hedgehog, FGF, Notch and PTHrP (Figures 3 and 4; Table 3).

### Crosstalk between TGF-β and BMP signaling with Wnt signaling

TGF-β cooperates with Wnt to stimulate both osteoblast and chondrocyte differentiation in a positive regulatory loop. TGF-β upregulates the expression of Wnts (Wnt-2, -4, -5a, -7a, and -10a) as well as Wnt co-receptor LRP5, and suppresses the expression of β-catenin inhibitor Axin1/2, to promote Wnt/β-catenin signaling.^194,195^ On the other hand, Wnt signaling induces Runx2-mediated TGFβRI expression in a β-catenin-independent way to promote TGF-β signaling.^196^ Bosch *et al*.^20^ showed that canonical Wnt signaling skews TGF-β signaling towards signaling via ALK1-Smad1/5/8 to promote chondrocyte hypertrophy.

BMPs have dual functions in regulating Wnt signaling. BMP induces Dkk1 and Sost expression through MAPK and Smad signaling, respectively, via BMPRIA to inhibit Wnt signaling in osteoblast, and negatively regulated bone mass.^96,98^ Smad4 competitively binds β-catenin and prevents its binding with Tcf/Lef transcription complex.^123^ Thus, ablation of Smad4 either in the crest neuron stem cell or in osteoblast results in upregulation of canonical Wnt signaling so as to promote bone formation.^60,123^ BMP2-induced formation of Smad1-Dvl1 complex also restricts β-catenin and inhibits its activity.^197^ On the other hand, BMPs stimulate osteoblast and chondrocyte differentiation synergistically with Wnt signaling. BMP-2 promotes canonical Wnt signaling by inducing Wnt3a, Wnt1, Lrp5 expression and inhibiting the expression of β-TrCP, the F-box E3 ligase was found responsible for β-catenin degradation in osteoblasts.^198,199^ Ablation of Smad4 in the pre-osteoblasts depletes LRP5 expression and impairs both BMP and Wnt signaling.^123^ BMP-2-induced LRP-5 expression also contributes to chondrocyte hypertrophy and osteoarthritis progress.^200^ Osteoblastogenesis is maximized in the presence of both BMP and Wnt signaling.^198,201,202^ Smad and TCF/LEF/β-catenin cooperatively complex with each other on the promoter to achieve the highest expression of osteoblast genes (Dlx5, Msx2, and Runx2).^202^ Moreover, activation of Wnt signaling by Wnt3a or overexpression of β-catenin/TCF4 promotes BMP signaling by stimulating BMP2 transcription.^203^

### Crosstalk between TGF-β and BMP signaling with FGF signaling

FGF and BMP signal synergistically promote osteoblast differentiation. BMP signaling is essential for FGF-induced osteoblast differentiation,^204^ and the osteogenic effect of BMP2 is also repressed in the absence of FGF2.^205^ FGF2 is responsible for BMP-induced nuclei translocation and accumulation of Runx2 and P-Smad1/5/8.^206^ FGF2, FGF9, and FGF18 induce while DN-FGFR reduces BMP2 expression.^204,207,208^ Disruption of the FGF2 in mice impairs the expression of BMP2 and reduces bone mass.^205^ However, FGF2 has a critical role in osteoblast proliferation but inhibits mineralization while BMP2 is instrumental in stimulating mineralization.^209,210^ Thus, besides cooperative functions, FGF2 and BMPs have different roles at different stages of osteoblast differentiation.

Antagonistic BMP and FGF pathways control the differentiation and proliferation rate of chondrocytes in the growth plate.^76,110^ Although BMP signal promotes chondrogenesis, FGF signal is a negative regulator of chondrogenesis and multiple mutations with constitutive activity of FGFR3 result in achondroplasia in humans.^211^ BMP signaling is required to inhibit activation of FGF signaling (for example, STAT and ERK1/2 activation) at least in part by inhibiting the expression of FGFR1.^110^ FGF signals are downregulated by ectopic BMP expression or Chordin/Noggin deficiency, and upregulated by CHL1 ectopic expression.^153,212^ Chondrocyte-specific activation of Fgfr3 in mice induced premature synchondrosis closure and enhanced osteoblast differentiation around synchondrosis associated with promoted BMP signaling, due to increased BMP ligand and decreased BMP antagonist expression.^211^ Chondrocyte-specific deletion of BMPRIA rescued the bone overgrowth phenotype observed in Fgfr3-deficient mice by reducing chondrocyte differentiation.^168^

### Crosstalk between TGF-β and BMP signaling with Hedgehog signaling

BMPs and Hedgehog signaling cooperatively regulate osteoblastogenesis in long bones.^213,214^ IHH is required for BMP-induced osteogenesis *in vitro.*^213^ Hedgehog-Gli activators direct osteo-chondrogenic function of BMPs toward osteogenesis in the perichondrium.^215^ SHH-Gli2 stimulates BMP-2 expression at transcription level to enhance osteoblast differentiation.^216,217^ BMP2 also induces expression of IHH and downregulates expression of patched 1 (PTC1), a negative regulator of Hedgehog signaling, during osteoblast differentiation in a positive feedback loop.^213^

During growth plate development, IHH-PTHrP regulatory loop keeps chondrocyte in the proliferation pool and favors elongation of long bones. Previous studies have shown that BMP signaling upregulates IHH–PTHrP signaling *in vivo*, and maintains a normal chondrocyte proliferation rate in parallel with IHH.^218,219^ Expression of IHH, PTC1, and PTHrP receptor (PPR) were decreased in *Smad4-*deficient growth plates, but were enhanced in CA-ALK2-, CA-BMPRIA-, or CA-BMPRIB-overexpressed chick limb bud.^108,120^ In cultured mouse limb explants, BMP2 application induces and Noggin antagonizes IHH expression.^218^ BMP6 also promotes IHH expression to favor chondrocyte hypertrophy.^220^ Furthermore, chondrocyte-specific Smad1/5 CKO mice develop chondrodysplasia and exhibit abnormal growth at the end of bones, probably owing to an imbalance in the crosstalk between the BMP, FGF, and IHH/PTHrP pathways.^213^ On the other hand, IHH also promotes chondrocyte hypertrophy independent of PTHrP via integrating with BMP and Wnt signaling.^221^ Chondrocyte-specific deletion of PTC1 upregulated BMP expression and BMP–Smad signaling.^222^ IHH deficiency downregulates BMP expression and reduces cranial bone size and all markers.^214^

In axial skeleton development, sequential SHH and BMP signals are required for specification of a chondrogenic fate in presomitic tissue.^223^ In this process, SHH promotes BMP expression, and initiates an Nkx3.2/Sox9 autoregulatory loop that is maintained by BMP signals to induce chondrogenesis.^223^ On the other hand, BMP activity negatively regulates SHH transcription and a BMP-SHH negative-feedback loop serves to confine SHH expression during limb development.^153,212^ SHH signal was impaired in the defected craniobone in the absence of Chordin and Noggin, the two BMP cognate binding partners.^155^

### Crosstalk between TGF-β and BMP signaling with Notch signaling

Notch activation favors BMP-induced osteoblast differentiation.^224,225^ Notch inhibition represses expression of BMP target genes.^226^ BMP-2 and TGF-β regulates expression of signaling proteins in Notch pathway (for example, Lfng, Hey1, and Hes1).^227^ Conditional deletion of Jag1, the Notch ligand in CNC, revealed altered collagen deposition, delayed ossification, and reduced expression of early and late determinants of osteoblast development during maxillary ossification, associated with dysregulation of BMP receptor expression in the Maxillary mesenchymal stem cell.

### Crosstalk between TGF-β and BMP signaling with PTH signaling

Osteoblast-specific Tgfbr2 CKO mice have increased bone mass which is caused by hyperactivition of PTH type I receptor (PTH1R) and could be rescued by disruption of PTH signaling by injection of PTH (7-34) or ablation of PTH1R.^43,228^ Mechanistic studies show that Tgfbr2 directly phosphorylates the PTH1R, which modulates PTH-induced endocytosis of both receptors and attenuates both TGF-β and PTH signaling *in vivo.*^43,228^ PTH enhances MSC differentiation into the osteoblast lineage through endocytosis of the PTH1R/Lrp6 complex, which enhances the BMPs-receptor binding affinicty.^229^ Positive regulation of PTHrP expression by TGFβ/BMP signaling can be observed in Smad4 mutant mice, Smad1/5 mutant mice and chick limbs overexpressing constitutively active BMP receptors. It has also been proposed that TGF-β promotes PTHrP expression.^22^ Both TGF-β and PTH inhibit terminal differentiation of chondrocyte in the growth plate, while BMPs promotes this process.^230^

Taken together, these studies demonstrate that TGF-βs and BMPs cooperate with other cytokines (Wnt, Hedgehog, FGF, Notch, and PTHrP) to regulate osteoblast and chondrocyte differentiation. Further studies will not only elucidate the specific interactions between those signaling pathways in bone, but also explore the potential of combined treatment for bone diseases by targeting multiple signaling pathways to achieve optimal outcome.

## TGF-β and BMP signaling in human diseases and clinical application

Given the important roles of TGF-β and BMP in chondrogenesis and osteogenesis, mutations in TGF-β and BMP signaling cause a wide range of skeletal disorders in human (Table 2). Not only do these studies reveal the roles of TGF-β and BMP signaling in bone formation and homeostasis, but also they decipher the molecular mechanisms and pathogenesis underlying related bone diseases and, most importantly, provide significant insights into the development of novel therapies.

### Fibrodysplasia ossificans progressiva (FOP; OMIM 135100)

FOP is a rare autosomal dominant disease characterized by progressive ectopic ossification of connective tissues and skeletal muscles. FOP is caused by activating mutations in BMP type I receptor Acvr1. Among them, R206H is the first characterized and most prevalent mutation, while other site mutations were also reported and contribute to the clinical variability and diverse severity of the disease (for example, R258S, L196P, and G328E).^91,92,231–233^

### Brachydactyly type A2 (BDA2; OMIM 112600)

BDA2 is characterized by hypoplasia of the second middle phalanx of the index finger and sometimes the little finger.^234^ Inactivating mutations of *Bmpr1b* gene (for example, I200K, R486K, and R486Q)^235,236^ or *GDF5* gene (for example, L441P and R380Q)^237,238^ were reported in BDA2 patients. Dathe *et al.*^239^ reported another genetic cause of BDA2: duplication of a regulatory element downstream of *BMP2* repressed BMP2 expression. GDF5 mutations were also found to be the cause of a number of cartilage disorders, including symphalangsism,^238^ chondrodysplasia,^240^ and osteoarthritis.^16^

### Myhre syndrome (MIM 139210)

Inactivated SMAD4 mutation causes Myhre syndrome, which is a developmental disorder characterized by short stature, short hands and feet, facial dysmorphism, muscular hypertrophy, deafness, and cognitive delay.^117,118^ Myhre syndrome patients carry an I500 mutation at *Smad4* that results in a stabilized but unfunctional mutant.^118,241^

### Noggin-mutation-related diseases

As the major BMP antagonist, mutation of Noggin in human causes multiple bone disorders. Missense mutations in BMP antagonist Noggin cause tyrosine kinase-like orphan receptor 2 (ROR2)-negative brachydactyly type B (BDB2; OMIM611377), which is characterized by hypoplasia/aplasia of distal phalanges in combination with distal symphalangism and fusion of carpal/tarsal bones^242^. Three missense mutations of Noggin were reported to cause Tarsal–carpal coalition syndrome (TCC; OMIM 186570), an autosomal dominant disorder characterized by short first metacarpals causing brachydactyly as well as fusion of the carpals, tarsals, phalanges, and humeroradial.^243^ Truncating mutation in the *NOG* gene in two families caused autosomal dominant stapes ankylosis with broad thumbs and toes, hyperopia, and skeletal anomalies (OMIM 184460).^244^ Heterozygous noggin mutations were reported to cause segregating proximal symphalangism (SYM1; OMIM 185800), which is characterized by ankylosis of the proximal interphalangeal joints, carpal and tarsal bone fusion or segregating multiple synostoses syndrome (SYNS1; OMIM 186500), which is characterized by multiple joint fusions.^138^

### Other human bone diseases caused by mutations in TGF-β/BMP signaling

Several mutations in the TGF-β1 gene were reported to cause Camurati–Engelmann disease (CED; OMIM131300), a rare autosomal dominant disease characterized by cortical thickening of the diaphyses of long bones, hyperostosis, and bone pain.^245,246^ Nonsense, missense, and frameshift mutations of Smad3 were reported to cause aneurysms–osteoarthritis syndrome (AOS; OMIM 613795), which is presented with aneurysms, dissections, and tortuosity throughout the arterial tree in association with mild craniofacial features as well as skeletal and cutaneous anomalies.^15,60,61^

Both BMP and TGF-β possess clinical implications to treat bone diseases including bone healing, rheumatoid arthritis *ect*. TβRI kinase inhibitor downregulates rheumatoid synoviocytes and prevents the arthritis.^247^ A small molecule inhibitor of BMP type I receptor activity has been demonstrated to be useful in treating FOP and heterotopic ossification syndromes.^248^ Currently, two BMP products have been approved by the Food and Drug Administration for clinical applications to treat fractures of long bones and improve intervertebral disk regeneration through a purified collagen matrix, respectively, infused with BMP-2 (Medtronic) or OP-1 BMP-7 (Stryker Biotech) and implanted at the site of the fracture.^4^ With the use of BMPs increasingly accepted in spinal fusion surgeries, other therapeutic approaches targeting BMP signaling are emerging beyond applications to skeletal disorders.^4^ In addition, GDF-5 has emerged as a therapeutic target for rheumatic diseases.^249^ Recent applications graft BMP peptides corresponding to residues 73–92, 89–117, and 68–87 of BMP-2, BMP-7, and BMP-9 as adhesion peptides (GRGDSPC) onto polyethylene terephthatalate surfaces to enhance osteogenic differentiation and mineralization of pre-osteoblastic cells.^250^ These engineered biomaterials for enhanced bone regeneration are in the initial trial stage of development.

To sum up, mutations in the BMP and TGF-β signaling-related genes result in multiple inheritable bone diseases in humans, including ossification disorders, joint diseases, and skeleton developmental defects. In the future, more molecules or peptides targeting BMP and TGF-β signaling will be developed to treat genetic bone disorders, fracture healing, and osteoarthritis diseases.

## Summary and perspective

BMP and TGF-β signaling pathways have important roles in skeletal development and postnatal skeleton homeostasis, by crosstalking with multiple signaling pathways, such as Wnt, Hedgehog, Notch, and FGF. Our understanding of BMP and TGF-β signaling is advanced with the generation of related mouse models, heritable human disease genetic studies and other new technologies including high-throughput screening. Specificity and versatility of BMP and TGF-β signaling are controlled by various ligand-receptor combinations, and transduced by co-Smad/R-Smad complex or MAPK cascade. The signaling is also dedicatedly controlled by factors including extracellular cognate binding proteins, I-Smad, epigenetic factors, and microRNA. Disruption of BMP and TGF-β signaling results in various bone disorders, such as osteoarthritis, FOP and Myhre syndrome. Manipulating BMP and TGF-β signaling pathways possess clinical implications for the treatment of multiple bone diseases including fracture healing, osteoarthritis, osteoporosis, and FOP. So far, BMP-2-and BMP-7-containing osteogenic implants have been used in over one million patients worldwide for the treatment of long bone nonunions, spinal fusions, and acute fractures. More importantly, with the aging population expected to double over the next decade, the number of patients suffering from osteoarthritis and osteoporosis is likely to increase dramatically and so is the cost of Medicare. Thus, it is compelling to elucidate the pathophysiology and the molecular mechanisms underlying skeleton health and bone diseases. Further, TGF and BMP signaling study will provide significant insights into the mechanism underlying how TGF and BMP signaling regulates osteoblast and chondrocyte proliferation, differentiation, maturation and activity in bone and cartilage formation, under both physiological and pathological condition (for example, osteoarthritis, osteoporosis, and bone cancer metastases. This study will also facilitate the design of novel therapeutic approaches for bone diseases.

## Figures and Tables

**Table 1:** Mouse models of TGF-β/BMP signaling in bone

Classification	Gene	KO/CKO/Tg	Defects	Reference
TGF-β ligands and receptors	*Tgfb1*	KO	Die at 1 month, normal skeleton	^25^
		*Tgfb1–/–Rag2–/–*	Evade early death, lower bone density	^8^
		*Col1* promoter; CA-TGFβ1 Tg	Defects resembling Camurati–Engelmann disease, osteoarthritis	^8,37^
	*Tgfb1/ Tgfb2*	DKO	Lack of distal parts of the rib	^251^
	*Tgfb2*	KO	Defects in both intramembranous bone and endochondral bone, craniofacial defects	^24^
	*Tgfb3*	KO	Normal skeleton, palate cleft	^27^
	*Tgfbr2*	*Col2-*Cre	Defects in the skull base and vertebrae, normal long bone	^47^
		*Prx1-Cre*	Short limbs and phalange joint fusion	^44–46^
		*Ocn-*Cre	Increase bone mass	^43^
		*nestin–*Cre^*TM*^	Less-developed osteoarthritis	^37^
		*MTII* promoter; DN-TβRII Tg	Bifurcation of the xiphoid process and sternum, automatic osteoarthritis	^34^
		*Dermo1-*Cre	Short and wide long bone, joint fusion, ectopic chondrocyte protrusion, reduced bone density	^30^
	*Alk5*	KO	Increase bone mass, decreased bone turn over, reduced body size, early ossification of the skull base	^39^
BMP ligands and receptors	*Bmp2*	*Col2-*Cre	Chondrodysplasia	^86^
		KO	Newborn death, skeletal patterning defects restricted to the rib cage, the skull, and the hindlimbs	^80^
	*Bmp7*	*Prx1-*Cre	Normal bone and no defects in fracture healing, progressive osteoarthritis	^70^
		*Prx1-*Cre	Mild defects in skeletogenesis, normal osteogenesis	^82^
	*Bmp2/7*	*Prx1-*Cre	Normal bone and no defects in fracture healing	^84^
	*Bmp4*	*Prx1-*Cre	Impairment of osteogenesis, malformed limbs	^82^
	*Bmp2/4*	*Col2-*Cre	Chondrodysplasia	^86^
	BMP-2/-4/-7	KO	More trabecular bone	^72^
	*Bmp3*	Tg	Less bone, defected bone collar, spontaneous rib fracture	^73^
		KO	Phalangeal defects	^77,109^
	*Bmpr1b*	*Col1* promoter; DN-BMPRII Tg	Reduced bone, delayed calvarial and vertebrae mineralization	^94^
		*Col2-*Cre	Chondrodysplasia, shortened bone, delayed ossification	^104,110^
	*Bmpr1a*	*Ocn-*Cre	Low bone mass, low bone turnover	^95^
		*Col1-*Cre;ER^TM^	Increase bone mass, more bone resorption, less OB	^96–98^
		*Gdf5-*Cre	Articular cartilage wears	^89^
	*Acvr1*	*Col2-*Cre	Defected skull and cervical vertebrae, progressive kyphosis	^105,252^
		CA-ALK2 Tg	Ectopic ossification resembling FOP	^90^
	*Bmpr1a/ Bmpr1b*	*Col2-*Cre	Absent skeleton elements formed through endochondral ossification	^104,110^
	*Acvr1/ Bmpr1a*	*Col2-*Cre	generalized perinatal lethal chondrodysplasia	^105^
	*Acvr1/ Bmpr1b*	*Col2-*Cre	generalized perinatal lethal chondrodysplasia	^105^
Smad pathway	*Smad1*	*Col2-*Cre	Defected calvarial bone development, shortening of growth plate	^113,114^
		*Col1-*Cre	osteopenia	^113^
	*Smad2*	KO	severe craniofacial defects	^253^
	*Smad1/Smad5*	*Col2-*Cre	Severe chondrodysplasia	^115^
	*Smad3*	KO	Osteopenia, accelerated chondrocyte hypertrophy, osteoarthritis	^52^
		*Col2-*Cre	progressive osteoarthritis	^58^
	*Smad4*	*Col2-*Cre	Dwarfism, disorganized growth plate, ectopic bone collars	^120^
		*Ocn-*Cre	Lower bone mass <6-month, more trabecular bone >7-month	^121^
		*Osx-*Cre	Stunted growth, spontaneous fracture, Osteogenesis imperfecta, CCD, Wnt-deficiency symdrome	^122^
		*Osx-*Cre	Increase mitosis, decrease differentiation & mineralization of OB	^123^
Non-Smad pathway	*TAK1*	*Osx-*Cre	Clavicular hypoplasia and delayed fontanelle fusion	^127^
		*Col2-*Cre	Cartilage defects, failure to maintain interzone cells of the elbow joint	^125^
		*Prx1-*Cre	Cartilage defects, widespread joint fusions	^125^
Other regulators	*Smad6*	*Col2* promoter; Tg	Dwarfism, osteopenia, delayed chondrocyte hypertrophy	^162^
	*Smad6/Smurf1*	*Col2* promoter; Tg	Severely delayed endochondral bone formation	^162^
	*Smad7*	*Prx1 promoter*; Tg	Impede condensation, poor cartilage formation	^165^
	*Smurf1*	KO	Age-dependent increase of bone mass	^167^
	*Tob*	KO	High bone mass	^174,175^
	*Ltbp-3*	*Prx1-*Cre	Normal bone but spontaneous fracture	^83^
	*Nog*	KO	Malformed skeleton, rescued by reduction of Bmp4 dosage	^138,139^
		*Ocn* promoter; Tg	Osteopenia	^142,143^
	*Nog/Grem1*	DKO	No sclerotome	^140^
	*Nog/Follistatin*	DKO	No sclerotome, trunk cartilage formation	^141^
	*Nog/Chordin*	*Chordin–/–Nog+/–*	Head defects	^155^

BMP, bone morphogenetic protein; TGF-β, transforming growth factor-β.

**Table 2:** TGF-β/BMP mutations involved in bone diseases

Gene	Disease	MIM	Bone defects	Reference
*Acvr1*	Fibrodysplasia ossificans progressive (FOP)	135000	Ectopic bone formation	^91,92,231–233^
*Bmp2*	Brachydactyly type A2 (BDA2)	112600	Hypoplasia of finger	^239^
*Bmpr1b*	Brachydactyly type A2 (BDA2)	112600	Hypoplasia of finger	^235,236^
*Gdf5*	Brachydactyly type A2 (BDA2)	112600	Hypoplasia of finger	^237,238^
	Symphalangsism		Joint disorder	^238^
	Chondrodysplasia, Greve type	200700	Severe abnormality of the limbs and limb joints	^240^
	Osteoarthritis, susceptibility	612400	Hip osteoarthritis	^16^
*Smad4*	Myhre syndrome	139210	Short stature, facial dysmorphism	^118,241^
*Nog*	Brachydactyly type B 2 (BDB2)	611377	Distal symphalangism, multiple joint fusion of distal bones	^242^
	Tarsal–Carpal coalition syndrome (TCC)	186570	Brachydactyly, multiple joint fusion of distal bones	^243^
	Stapes ankylosis with broad thumbs and toes	184460	Stapes ankylosis with broad thumbs and toes, hyperopia, and skeletal anomalies	^244^
	Segregating proximal symphalangism (SYM1)	185800	Multiple joint fusion of distal bones	^138^
	Segregating multiple synostoses syndrome (SYNS1)	186500	Multiple joint fusion of distal bones	^138^
*Tgfb1*	Camurati–Engelmann disease (CED)	131300	Osteosclerosis affecting diaphysis of long bone, hyperostosis, bone pain	^245,246^
*Smad3*	Aneurysms osteoarthritis (AOS)	613795	Mild craniofacial feature, skeletal anomalities, osteoarthritis	^15,60,61^

**Table 3:** Crosstalk between TGF-β/BMP signaling and other signaling molecules in bone

Gene	Crosstalk signaling	Results	Reference
TGF-β 1→	β-catenin stability↑	Osteoblastogenesis↓	^254^
TGF-β 1→	Wnts↑, LRP5↑, Axin1/2↓	Chondrocyte differentiation↑, adipocyte differentiation↓	^194,195^
Wnt	TGF-β-ALK5-Smad2/3 **→**TGF-β-ALK5-Smad1/5/8	Chondrocyte hypertrophy↑	^20^
Smad4↓	LRP5↓, β-catenin activity↑	Bone formation↑	^60,123^
BMP-2→BMPR1A→	SOST↑, Dkk1↑**→** β-catenin ↓	Bone mass**↓**	^96,98^
BMP-2	Lrp5↑, Wnt3a↑, Wnt1↑, β-TrCP↓**→**β-catenin ↑	Osteoblast differentiation↑, chondrocyte hypertrophy↑	^198–200^
BMP and Wnt	Smad complex with TCF/LEF/β-catenin	Osteoblast differentiation↑	^201,202^
Wnt3A→	↑BMP-9, ↑BMP-2	↑ALP	^201,203^
TGF-β1→	↑BMP-2	→Ectopic bone formation	^255^
FGF2↑	Tgfbr2 mutant→normal	Regulates frontal bone	^256^
FGF-FGFR3	TGF-β	Mediates embryonic bone formation	^256^
FGF-2,-9 →FgfR→	↑BMP-2 and TGF-β 1	↑Osteogenic expression	^208^
FGF-2→	BMP-induced osteogenesis↑	Osteoblast differentiation↑	^205,206^
BMP-2→	FGF-induced osteogenesis↑	Osteoblast differentiation↑	^204^
BMP and FGF	Antagonized function in the growth plate	Balance chondrocyte differentiation and proliferation	^76,110^
BMPRIA↓	Rescue over-growth of Fgfr3−/− mice	Balance chondrocyte differentiation and proliferation	^168^
Notch	↑BMP-induced ALP	→Smad and Notch	^225^
SHH (Gli2) →	↑BMP-2	Normal osteoblast differentiation	^217^
IHH→	↑BMP-induced osteogenesis	Bone formation	^213^
IHH and BMP→	↑ALP, ↑IHH	Long bone development	^257^
Tgfbr2↓	↑PTH type I receptor activity	Increased bone mass	^43^
PTH→CREB→	↑BMP-2	Osteoblastogenesis	^258^

↓decrease; ↑increase; →stimulate.

BMP, bone morphogenetic protein; FGF, fibroblast growth factor; IHH, Indian hedgehog; PTH, parathyroid hormone; SHH, Sonic hedgehog; TGF-β, transforming growth factor-β.
